# Investigating Eye Contact Effect on People’s Name Retrieval in Normal Aging and in Alzheimer’s Disease

**DOI:** 10.3389/fpsyg.2019.01218

**Published:** 2019-05-29

**Authors:** Desirée Lopis, Laurence Conty

**Affiliations:** Laboratory of Human and Artificial Cognition (CHArt EA4004), Paris Nanterre University, Nanterre, France

**Keywords:** face-name memory, associative memory, eye contact effects, aging, Alzheimer’s disease

## Abstract

Difficulty in recalling people’s name is one of the most universally experienced changes in old age and would also constitute one of the earliest symptom of Alzheimer’s disease (AD). Direct gaze, i.e., another individual’s gaze directed to the observer that leads to eye contact, has been shown to improve memory for faces and concomitant verbal information. Here, we investigated whether this effect extends to memory for Face-Name association and can thus enhance names’ retrieval in normal aging and in AD, at the early stage of the disease. Twenty AD patients, 20 older adults and 25 young adults participated in our study. Subjects were presented with faces displaying either direct or averted gaze in association with a name presented orally. They were then asked to perform a surprise recognition test for each pair of stimuli, in a sequential fashion (i.e., first categorizing a face as old or new and then associating a name using a forced-choice procedure). Results showed that direct gaze does not improve memory for Face-Name association. Yet, we observed an overall direct gaze memory effect over faces and names independently, across our populations, showing that eye contact enhances the encoding of concomitantly presented stimuli. Our results are the first empirical evidence that eye contact benefits memory throughout the course of aging and lead to better delimit the actual power of eye contact on memory.

## Introduction

A global decline in memory abilities is commonly observed in aging (e.g., Craik and Rose, 2012; Nyberg et al., 2012), with episodic memory being particularly affected (e.g., Cansino, 2009; Tromp et al., 2015). Difficulty in recalling people’s name is one of the most universally experienced changes in old age and a common everyday cognitive complaint of elderly individuals (e.g., Reese et al., 1999; Cargin et al., 2008). In some questionnaire studies on everyday memory ability, this difficulty to recall names is “*singled out by many of the elderly respondents as the most noticeable and most frustrating change in cognitive ability”* (Cohen and Faulkner, 1984, p. 50). Previous studies suggest that older adults show difficulties in naming pictured celebrities more often than young adult (e.g., Maylor, 1990; Cross and Burke, 2004), and that age-related decline is larger for producing proper names than common nouns (Evrard, 2002; Rendell et al., 2005). Because names must be both remembered and mentally associated to the corresponding faces, this difficulty would mostly rely on deficits in associative memory, i.e., the memory for associations between concomitantly presented information (Naveh-Benjamin et al., 2004; Old and Naveh-Benjamin, 2008). The associative deficit hypothesis (ADH) thus “*attributes age-related declines in associative memory to older adults’ inability to encode and retrieve the relationships between single units of information*” (Naveh-Benjamin et al., 2009, p. 221).

Forgetting names is also the most common early symptom of Alzheimer’s disease (AD) (Irish et al., 2011; Tak and Hong, 2014). This is not surprising since the underlying neuroanatomical structures supporting the associative memory is the hippocampus which is known to be affected early in the process of AD (Erk et al., 2011). Also, in healthy individuals, face-name associative memory performance was found to be inversely correlated with amyloid burden (i.e., one of the central neuropathological features of AD) in brain region associated with memory systems (Rentz et al., 2011), leading the authors to suggest that this type of memory might be a sensitive marker to detect preclinical stages of AD. Moreover, the ability to remember names and faces decreases concomitantly with the progression of the disease (Tak and Hong, 2014), eventually leading a person with AD to not only forget peoples’ names but also to ignore the identity of the person she or he is talking to. This results in experiencing shame and loss of self-confidence, eventually promoting social withdrawal among persons with AD.

Human faces convey critical socio-emotional signals in everyday life. Gaze, in particular, plays a critical role in the regulation of inter-individual exchanges (Kleinke, 1986). Among all gaze directions, it has been shown that direct gaze, i.e., another individual’s gaze directed to the observer that leads to eye contact, implicitly influences a wide range of cognitive processes and behaviors (Senju and Johnson, 2009). Among all the so-called “Watching eyes effects” (W.E. effects, Conty et al., 2016), direct gaze has been robustly shown to increase memory for face identity in young adults (Hood et al., 2003; Vuilleumier et al., 2005; Conty and Grèzes, 2011). However, the use of eye contact during interaction not only improves memory for faces but would also enhance memory for concomitant verbal information. For example, results from two different studies conducted in a school context converge in showing that students remember more instructions or details from a story when the teacher gaze at them more frequently (Fry and Smith, 1975; Ottenson and Ottenson, 1979). Fullwood and Doherty-Sneddon (2006) also found positive effects of mutual gaze upon verbal information recall: establishing eye contact with the listener during verbal presentations improves memory for verbal content, compared to presentations delivered with no mutual gaze.

Altogether, these findings point toward the possibility that direct gaze facilitates the face-name association retrieval. If true, eye contact could be used during interactions to compensate the difficulty in recalling names emerging in normal aging and in AD (Baltazar, 2015; Conty et al., 2016). To the best of our knowledge, this hypothesis has never been tested. The purpose of the present study was to investigate the existence of the W.E. effects on memory for face-name association retrieval in healthy young adults, older adults and in AD patients at the early to mild stage of the disease.

It has been argued that the W.E. effects rely on a unique self-referential mechanism, suggesting that they should evolve together during lifespan (see Conty et al., 2016 for more details). Yet, there is a lack of data about the persistence of these effects in normal and pathological aging. We recently started to investigate this issue by focusing on the impact of direct gaze on the modulation of others’ appraisal (Kleinke, 1986; Kuzmanovic et al., 2009) and on memory for faces in healthy young adults, healthy old adults and in old adults with AD (Lopis et al., 2017). We exposed participants to faces with different eye directions (direct vs. averted) and asked them to rate each face’s degree of likeability. Participants were then asked to identify the previously seen faces during a surprise recognition test. The results revealed for the first time that the effect of direct gaze on other’s appraisal (i.e., rating faces with direct gaze as more likeable than faces with averted gaze) is preserved in normal aging as well as until the mild stage of AD. However, the effect on memory for faces emerged exclusively in young participants. On one hand, these findings are encouraging as they showed that some of the W.E. effects still emerge in normal and pathological aging. On the other hand, this invalidated the self-referential model of the W.E. effects, thus calling for further investigations. In our previous study, one explanation for the absence of memory effect of eye contact in older adults – with or without AD – can rely on the lack of salience of the face stimuli.

In our previous study, we used standardized digital grayscale portraits as facial stimuli. Yet, increasing environmental support can improve memory (Craik et al., 1986). More specifically, Bender et al. (2017) recently showed that, regardless of the observer’s age, highly distinctive, colored face stimuli and accompanied by multiple non-facial details, as well as salient characteristics such as eye color and head rotation, are easier to recognize and require shorter processing time than less distinctive, grayscale face stimuli. Thus, enriching stimuli by adding external contextual cues – namely, keeping the colored version of the portraits and attributing names to the individuals – may be a plausible way to enhance their ecological validity and therefore increase their salience for older adults.

We thus exposed participants to colored pictures of unfamiliar faces with different eye directions (direct versus averted). Concomitantly to each face presentation, participants heard the name of the individual portrayed in the picture. We asked them to make a decision about whether the name “fitted” or “fitted very well” the face it was associated to Naveh-Benjamin et al. (2009). After an interfering task, participants were submitted to a two-stage surprise recognition test; they were first asked to recognize the previously seen faces, then their names. We predicted that the face-name associations assigned to individuals initially displayed with a direct gaze would be better recognized compared to the associations initially assigned to individuals gazing away from the participant. We recruited AD patients, matched older participants without cognitive impairment and healthy young subjects, in order to distinguish effects pertaining to normal or pathological aging.

## Materials and Methods

### Participants

A total of 65, right-handed, native French-speaking participants were included in the study: 20 patients with a diagnosed AD (14 women; mean age ± standard deviation = 81.8 ± 5.8 years), 20 community-dwelling healthy older adults (OA, 14 women; mean age = 79.9 ± 4.8 years) and 25 healthy young adults (YA, 12 women; mean age = 22.8 ± 3.4 years). We based our sample size calculation on the previous results obtained in our groups regarding the W.E. effects (Lopis et al., 2017). The minimum effect size f in these studies was 0.62 (i.e., $\eta_{p}^{2}$ = 0.28). Based on this value, we computed a total sample size of 45 for a power of 0.9, at alpha 0.05 using the software G^∗^Power 3 (Faul et al., 2007). We then recruited 25 participants for each group with the aim of accounting for potential exclusion of participants due to technical errors or meeting of exclusion criteria (i.e., neuropsychological tests’ or depression scores, see details below). One AD patient was excluded because he did not meet the GDS maximum criterion score of 6. Two older adults were also excluded: one who did not meet the MMSE minimum criterion score of 26 and another for technical issues in collecting the data. Lastly, four supplementary AD patients and three supplementary OA had to be excluded in order to meet the matching criteria for age, gender distribution, years of education and level of depression between the AD patients and OA groups. None of the young participants was excluded.

Young adults were recruited by advertisements spread on a French internet database of volunteers willing to participate in psychology or neuroscience research. OA were community dwelling and were recruited by advertisements and notices distributed through senior citizen organizations in the Paris areas. The patients with AD were recruited from a local memory center and were at the early to mild stage of the disease (MMSE between 19 and 24; Feldman and Woodward, 2005). All participants had normal or corrected-to-normal vision and were naive to the aim of the experiment. They provided written informed consent according to institutional guidelines of the local research ethics committee (who stated on the compliance with the Declaration of Helsinki). The whole procedure was approved by the local ethics committee (*Comité de Protection des Personnes* Ile-de-France-X, protocole 02^(2)^ 2015 – ALCOM No. 2014-A01141-46).

All participants underwent structured interviews and neuropsychological testing to assess cognitive functioning. A full description of the groups of participants is presented in Table 1. The diagnosis of probable or possible AD was assigned to patients by a neurologist according to the criteria of the National Institute of Neurological and Communicative Disorders and Stroke and the Alzheimer’s disease and Related Disorders Associations (NINCDS/ADRDA; McKhann et al., 2011). AD patients were excluded if they were judged to be unable to understand task instructions. None of the AD patients was reported to have prosopagnosia. For controls, the following exclusion criteria were applied: history of neurological disorders, traumatic brain injury with loss of consciousness and significant history of psychological or psychiatric disorders.

**Table 1:** Means and SDs of demographics, general neuropsychological efficiency and depression scores.

	Young adults (YA)	Older adults (OA)	AD patients	Differences between YA and OA		Differences between OA and AD patients	
				*t*-Value	*p*-Value	*t*-Value	*p*-Value
*N* (F:M)	25 (12:13)	20 (14:6)	20 (14:6)	– ^a^	n.s.	– ^a^	n.s.
Age (years)	22.8 (3.4)	79.9 (4.8)	81.8 (5.8)	−46.2	<0.000	−1.1	n.s.
Level of education (years)	13.3 (2.3)	11.9 (4.6)	10.2 (4.1)	1.3	n.s.	1.1	n.s.
General cognitive efficiency MMSE (30) ^b^	28.2 (1.2)	27.9 (1.0)	21.6 (2.7)	0.9	n.s.	9.5	<0.000
Frontal efficiency FAB (18) ^b^	16.8 (1.1)	16.1 (1.3)	13.6 (2.3)	1.8	0.06	4.1	<0.000
Episodic memory 5-words test (10)^b^	10.0 (0.0)	9.3 (0.9)	6.2 (2.5)	3.8	<0.000	4.9	<0.000
Attention and working memory Forward digit span	6.5 (0.9)	5.1 (1.0)	5.0 (1.0)	4.6	<0.000	0.3	n.s.
Backward digit span	4.8 (1.2)	3.4 (0.9)	3.8 (0.7)	4.1	<0.000	−1.6	n.s.
Depression GDS (cut-off < 7/15)	– ^c^	1.8 (1.9)	1.4 (1.4)	– ^c^	– ^c^	0.7	n.s.

*Two-tailed t-tests for independent samples were used. SD, standard deviation; n.s., not significant; AD, Alzheimer’s disease; MMSE, Mini Mental State Examination; FAB, Frontal Assessment Battery; GDS, Geriatric Depression Scale. ^a^Gender distribution across groups was tested by using a two-sided Fisher exact test for Count Data. ^b^Maximum possible score. ^c^Not applicable. Young adults were screened for present major depression by using the Mini International Psychiatric Interview 5.0.0 (French version, Lecrubier et al., 1998).*

The neuropsychological evaluation consisted in exploring global cognition with the Mini Mental State Examination (MMSE; Folstein et al., 1975), frontal lobe and executive functions with the Frontal Assessment Battery (Dubois et al., 2000), episodic memory with the 5-words test (Dubois et al., 2002), attention and working memory with the forward and backward digit spans (Wechsler, 1997). As for psychiatric evaluation, older participants (OA and AD patients) fulfilled the 15-items Geriatric Depression Scale (Yesavage et al., 1983) and those who scored 7 or more on this scale were excluded from the study. The Mini International Psychiatric Interview 5.0.0 (French version, Lecrubier et al., 1998) was administered to YA to screen for present major depression.

All healthy participants had performances within the normal range in all neuropsychological screening tests (i.e., having a score no more different than 1.65 SD compared to the mean of their group of reference, as provided in the norms of each test). All healthy OA had an MMS score superior or equal to 26. None of them expressed any complaints about their memory. All were paid for their participation.

Older adult and AD groups were matched for age, gender distribution, years of education, and level of depression (see details in Table 1).

### Stimuli

#### Facial Stimuli

Seventy static colored photographs of 30 individuals (15 men/15 women) were selected from a database of digitized portraits of adult faces (see Vuilleumier et al., 2005; Conty et al., 2007). All faces had neutral expression and involved individuals unknown to our participants. The age of each individual ranged from 20 to 60 years and our stimuli selection included approximately 1/3 of young-looking faces, 1/3 of middle-aged-looking faces, and 1/3 of old-looking faces. Head direction was always oriented straight toward the observer. All the 30 individuals were photographed with closed eyes. Twenty individuals out of 30 (10 men/10 women) were randomly selected and considered as Target-Faces. They were available in two supplementary views: one with the eyes directed straight toward the observer (Direct Gaze condition), one with the eyes averted by 30° toward the right side from the observer position (Averted Gaze condition). Face stimuli with averted gaze were mirrored to obtain both left-averted and right-averted gaze pictures. Two sets of these 20 Target-Faces were created, F1 and F2. On each set, half of the individuals were shown with direct gaze and the other half with averted gaze (right-averted for half of the participants and left-averted for the other half). The association between Target-Faces and gaze direction compiled for the set F1 was reversed for the set F2. The 10 remaining individuals constituted a set of “New” Faces.

#### Name Stimuli

Forty name (20 males/20 females) were extracted from the French National Institute of Statistics and Economic Studies website^1^, with a specific tool allowing to select the most frequently attributed names to children born in the birth cohorts from 1960 to 2000, ranked on popularity. Each selected name was uttered by a female voice and recorded by a professional Digital Voice recorder. The volume was normalized with the AUDACITY 2.0.6 freeware. Two sets of 20 names were created (10 males/10 females): a set of Target Names and a set of New Names. The two sets matched in terms of duration (ranging from 600 to 900 ms), frequency (medium frequency of occurrence of 10,000 to 30,000 times in France between 1960 and 2000) and phonetic properties (same number of syllables, with no more than three syllables), overall and across gender.

### Procedure

Participants sat approximately at 70 cm in front of a Dell computer with a 15.6 inches screen (with a resolution of 1366 × 768 pixels) on which face stimuli were shown on a black background. The name stimuli were presented through speakers plugged to the computer. E-Prime^®^ 2.0 software was used to control stimulus presentation, response recording, and latency (Schneider et al., 2002).

**FIGURE 1:**
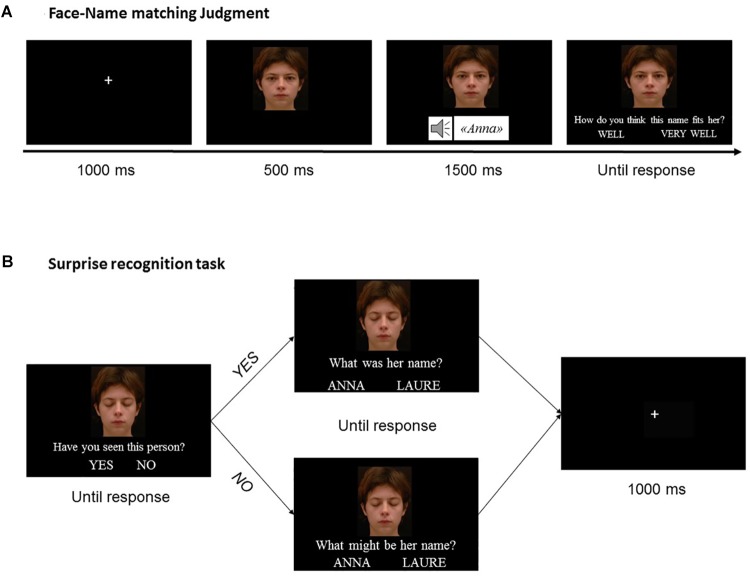
Illustration of the experimental design. **(A)** Illustration of an experimental trial of the Study Phase. During the study phase, subjects were presented with faces displaying either direct or averted gaze in association with a name presented orally and performed a Face-Name matching task (i.e., they indicated whether the name fitted “*Well*” or “*Very well*” the face it was associated to). **(B)** Illustration of an experimental trial of the Test Phase. After a 5-min interfering task, participants were asked to perform a surprise recognition test for each pair of stimuli, in a sequential fashion (i.e., the face first, then the name). They were asked to perform an Old-New recognition task over faces, followed by a forced-choice recognition task over names. As for name recognition, two possible questions could be displayed, depending on participants’ previous answer (whatever it was correct or not). If they indicated that they actually recognized the face shown on the screen, they were asked “*What was his/her name?”* If they reported to never have seen the face before, the displayed question was “*What might be his/her name?”* Written informed consent was obtained from the depicted individual presented in **(A,B)** for the publication of his identifiable images.

The experiment was divided into three parts: an initial encoding task (Study phase), a 5-min interfering phase and a surprise recognition task (Test phase) (Figure 1). Four versions of the experiment, corresponding to different 20 Target Face-Target Name pairs were created. Two of them with the set of 20 Target-Faces F1, the other 2 with the set of 20 Target-Faces F2. The 4 versions were created using the set of 20 Target Names. In each version, each Target Name was randomly associated with a Target Face, with the following constraints: (a) face-name gender matching, (b) across the versions, each Target Name was associated with a different Target Face, 2 of them presented with direct gaze and the other 2 with averted gaze. Each participant processed only one version of the experiment, and the four versions were equally processed across participants.

#### Study Phase

During the initial Study phase, participants were presented with 20 different Target Name-Target face pairs (i.e., 20 trials), one by one, in a randomized order. For each face, participants were asked to perform a forced-choice decision about whether the name “fitted” or “fitted very well” the face it was associated to (Naveh-Benjamin et al., 2009; Carr et al., 2017). We choose this incident task to favor the association between the two components, i.e., the face and the name. Participants were told that there was no correct or wrong answer for this task and that the aim was to examine subjective perception of how names and faces match together. Choices were made via a key press system (see details below).

Each trial started with a 1000 ms presentation of a fixation cross (visual angle of 2° × 2°) which was located at the level of the to-be-presented face’s eyes. Then the Target Face appeared on the screen, covering a visual angle of approximately 13° horizontally and 16° vertically. After 500 ms, the face remained displayed on the screen while the Target Name was presented orally. 1500 ms after the start of name presentation, the question “*How do you think this name fits him/her*?” along with the option choices’ tags “*Well*” and “*Very well*” appeared on the screen, under the face. The response cannot be provided before the appearance of the question, so that each individual’s face was seen during 2000 ms by each participant before she provided her response. The face remained on the screen until a response was given via a key press system (see details below). Immediately after the participant’s response, a black screen was displayed during 1000 ms, preceding the fixation cross of the next trial.

#### Interfering Task

After the Study phase, an approximately 5-min Interfering phase followed. Participants were submitted to a 3 min counting backward task, a commonly used interfering task during episodic memory evaluation (Wechsler, 1945; Folstein et al., 1975; Grober et al., 1987). They were asked to begin with 150 and count backward by 3 until 0. This interfering phase was directly followed by a surprise recognition test for each face-name pair (Test phase).

#### Test Phase

During the Test phase, the recognition of the face and of the associated name were processed in a sequential fashion. Participants were first asked to perform an Old-New recognition task on faces, i.e., to say, for each presented face, if they thought they have seen it before or not.

Participants saw a total of 30 faces with closed eyes (i.e., 30 trials), among which 20 “OLD” faces, i.e., the Target Faces previously seen by the participant during the Study phase, and 10 “NEW” faces, i.e., distractor-faces unknown to the participant (see Smith et al., 2006 for similar procedure). Closed eyes were used in order to specifically test the recognition of the identity of the face and to prevent participants from doing a superficial picture-matching task (Bruce, 1982). The order of face presentation was random. Each trial started with the presentation of a fixation cross during 500 ms at the center of the screen. Then, an individual’s face with closed eyes appeared on the screen, covering a visual angle of approximately 13° horizontally and 16° vertically. Such stimulus could be either an “OLD” face or a “NEW” face. A dialog box was concomitantly displayed, reporting the question “*Have you already seen this person*?” along with the response boxes “*Yes*” and “*No.*” Once the response was entered (see below for details), participants were then asked to perform a recognition task over names. For this second task, we had the choice to employ either a free recall paradigm (see, for example, Amariglio et al., 2012) or a forced-choice decision task in which the participant has to choose among two presented names, the one belonging to the face shown (see Polcher et al., 2017 for a similar procedure). We choose the latter to avoid floor effects in participants with cognitive deficits. In addition, this response format may also reduce potential frustration in older adults. Lastly, we did not employ the commonly used paradigm to investigate the ADH in elderly (i.e., submitting the participants to three different memory tests, two for the components and one for their associations, sequentially (Naveh-Benjamin et al., 2004, 2009) because of its duration and redundancy, which may have caused early fatigability in AD participants.

Concretely, as the face was still on the screen, another dialog box appeared, displaying two possible questions, depending on participants’ previous answer (whether it was correct or not). If they indicated that they actually recognized the face shown on the screen, they were asked “*What was his/her name?”* If they reported to never have seen the face before, the displayed question was *“What might be his/her name?”* Response boxes always displayed two possible names. If the face was an “OLD” one (whatever the response given by the participants), participants were given the choice between the Correct Name (i.e., the name with which the face was actually associated during the Study phase) and a Distractor Name which was always an “OLD’ name as well, but previously associated with direct gaze (50% of the trials) or with averted gaze (50% of the trials) and that was of the same gender as the target face. Thus, in half of the trials the Correct Name and the Distractor Name were associated with the same gaze direction during the Study Phase (Distractor Name – Same) while in the other half they were associated with different gaze direction (Distractor Name – Opposite). Distractor Name – Same were pre-selected, but changed for each of the four versions of the experiment. If the face was a “NEW” one, a choice was given to participants between two “NEW” names (i.e., stimuli that participants never heard of). These new names were randomly selected from the set of 20 New Names (see section “Stimuli”), with the following constraints: face/name gender matching; each name only seen once during the Testing phase. Once the response was entered (see below for details), a black screen appeared during 1000 ms, then the next trial begun.

#### Participants’ Response

In each experimental phase, dialog boxes were always displayed on the screen, concomitantly and under the face. Each item of the pair of response boxes (“Well/Very well,” “Yes/No,” “Correct Name/Distractor Name”) was always located respectively on the left and on the right side of the screen. The place of each item was fixed except for the Correct Name, which was randomly located, with the constraint to appear half of the cases on the left side of the screen for every participant. Participants were asked to answer all the questions by using a two-choice button press. A cover placed on the computer keyboard allowed the participants to only use two keys to enter their response: one located on the left side keyboard and the other one located on the right side.

#### Debriefing Interview

At the end of the experiment, participants were asked whether any of the faces used in the experiment was previously known to them and if they anticipated the incoming surprise recognition task during the Study phase. They were also asked to report any feeling of inconsistency regarding the face-name associations. All participants confirmed that none of the faces was familiar prior to testing. None of them pointed out the presence of inconsistent or unusual face-name pairs, nor did they anticipate the subsequent recognition task.

### Statistical Analysis

#### Demographic and Neuropsychological Data

Twenty AD patients, 20 healthy OA, and 25 YA were included in the analyses. In order to examine group differences in the total sample (*N* = 65), we applied a two-sided Fisher exact test for Count Data for categorical and analyses of variance (ANOVAs) for continuous variables, respectively. Following the ANOVA, we performed planned comparisons by using bilateral Student’s *t*-test when main effects or interactions were observed (significance level < 0.05).

#### Variables of Interest

We conducted repeated measures ANOVAs with Gaze Direction (direct/averted) as within-subjects factor and Group (AD/OA/YA) as between-subjects factor on the following variables of interest: mean time of exposure to the faces during the study phase (TEx, i.e., response time to the task + 2000 ms corresponding to the minimal exposure time), percentage of correct face recognition (Hits for faces) and associated RTs (*RTs of hits for faces*) and percentage of correct name recognition following OLD face correct recognition (Hits for complete face – name recognition, which was considered as the overt evidence of a successful Face-Name association). We also computed the percentage of correct name recognition following OLD face presentation, independently from its correct recognition (Hits for Name recognition) and associated RTs (RTs of Hits for Name recognition). This third score included all the trials where participants either recognized the OLD face or not, but still picked the correct associated name. We therefore assumed that it may still reveal that the association between the face and the name is successful, even if the face has not been systematically, explicitly recognized. This last variable was submitted to an ANOVA with Gaze Direction (direct/averted) and type of Distractor Name (Same or Opposite, see section “Materials and Methods”) as within-subjects factor and Group (AD/OA/YA) as between-subjects factor.

Partial Eta-squared ($\eta_{p}^{2}$) are reported as effect size indexes. As suggested by Cohen (1988), we considered effect sizes as being small for $\eta_{p}^{2}$ < 0.06, medium for 0.06 ≤ $\eta_{p}^{2}$ < 0.14, and marked for $\eta_{p}^{2}$ ≥ 0.14. For significant comparisons, Cohen’s d was used to determine effect size with *d* < 0.3 corresponding to a small effect, 0.3 < *d* < 0.8 to a medium effect and *d* > 0.8 to a large effect (Cohen, 1988).

Lastly, we computed the mean d’ parameter and the decision criterion C for each group of participants in order to assess groups’ discrimination performances and control for biased response criteria respectively (Green and Swets, 1966). This was done exclusively for the Old-New recognition task for faces since the forced choice decision paradigm we employed to test name recognition does not allow response bias computation.

In AD group, two participants didn’t recognize any “OLD” face during the Test phase. The computation of the Hits for complete face-name recognition and for Name Recognition were therefore impossible for them, so they were excluded from this analysis.

## Results

Descriptive statistics of the three groups are listed in Table 1.

### Study Phase

#### Time of Exposure to the Face (TEx)

The ANOVA with Gaze Direction as within-subjects factor and Group as between-subjects factor performed on the TEx (i.e., 2000 ms + response time, see section “Materials and Methods”) revealed a main effect of Group (*F*_(2,62)_ = 28.52; *p* < 0.0001; $\eta_{p}^{2}$ = 0.47). Planned comparisons showed that AD patients were slower in performing the task than healthy OA (respectively: mean = 5331 ± 1388 vs. 3839 ± 798 ms, *t*_(38)_ = 4.16; *p* < 0.001, *d* = 1.3), who were slower than YA (mean = 3213 ± 538, *t*_(43)_ = 3.13; *p* = 0.003, *d* = 0.9). Crucially, no effect of Gaze Direction, or interaction between Gaze Direction and Group, was found on this variable (all *p*_s_ > 0.1), indicating that all participants were exposed during the same amount of time to faces with direct and averted gaze.

### Test Phase

#### Hits for Faces

As expected, AD patients showed poorer discrimination performances (*d*′ = 0.33) than OA (0.57) who, in turn, showed poorer discrimination performances than YA (*d*′ = 1.60). Moreover, the decision criterion C was centrally placed for each group of participants (ranging from 0.00 to 0.09), showing that groups’ response criteria were not biased. These results supports the relevance of analyzing Hit rates as a sensitivity index (Azzopardi and Cowey, 1998).

**FIGURE 2:**
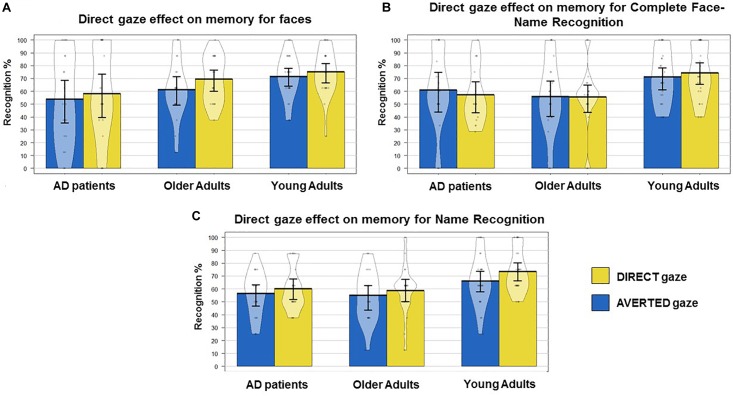
Young adults (YAs), healthy older adults (OAs) and AD patients’ behavioral results. Mean, standard error to the mean, raw data and the density curve showing the full data distribution are reported. **(A)** Percentage of recognition for faces presented with direct and averted gaze. A main effect of Group (*p* = 0.05) and a main effect of Gaze Direction (*p* = 0.03) are observed. Significant differences detected by planned comparisons: YA vs. OA and AD, trend for OA vs. AD patients. **(B)** Percentage for Complete Face-Name Recognition. A main effect of Group (*p* = 0.01) is observed. Significant differences detected by planned comparisons: YA vs. OA and AD. **(C)** Percentage of recognition for names. A main effect of Group (*p* = 0.007) and a trend for a main effect of Gaze Direction (*p* = 0.06) are observed. Significant differences detected by planned comparisons: YA vs. OA and AD.

The ANOVA with Gaze Direction as within-subjects factor and Group as between-subjects factor performed on the Hits for faces revealed a main effect of Group on this variable (*F*_(2,62)_ = 3.14, *p* = 0.05; $\eta_{p}^{2}$ = 0.09). Planned comparisons revealed that YA (mean = 76 ± 9%) recognized significantly more faces than OA (mean = 63 ± 9%, *t*_(43)_ = 4.46; *p* < 0.0001, *d* = 1.3) and AD (mean = 56 ± 14%, *t*_(43)_ = 5.51; *p* < 0.0001, *d* = 1.7). However, OA only showed a tendency to perform better than AD patients (*t*_(38)_ = 1.86; *p* = 0.06, *d* = 0.6). We also observed a main effect of Gaze direction (*F*_(1,62)_ = 4.89, *p* = 0.03; $\eta_{p}^{2}$ = 0.07), showing that, overall, participants recognized significantly more faces initially displayed with direct (mean = 67 ± 25%) than averted gaze (mean = 62 ± 26%) (Figure 2A). There was no interaction between Gaze Direction and Group (*F*_(2,62)_ = 0.34; *p* > 0.1).

#### Complete Face – Name Recognition

The ANOVA with Gaze Direction as within-subjects factor and Group as between-subjects factor performed on the percentage of complete face-name recognition (i.e., hits for OLD faces and associated names) revealed a main effect of group (*F*_(2,59)_ = 4.72, *p* = 0.01; $\eta_{p}^{2}$ = 0.13). Planned comparisons showed that YA (mean = 71 ± 15%) reached greater complete face/name recognition than healthy OA (55 ± 20%, *t*_(43)_ = 2.95; *p* = 0.005, *d* > 0.9) and AD patients (mean = 56 ± 23%, *t*_(41)_ = 2.55; *p* = 0.01, *d* > 0.7). There were no differences between healthy OA and AD patients (*t*_(36)_ < 1 – Figure 2B). There was no overall effect of gaze direction, or interaction between Gaze Direction and Group (all *t* < 1; all *p* > 0.1).

#### Hits for Name Recognition

**FIGURE 3:**
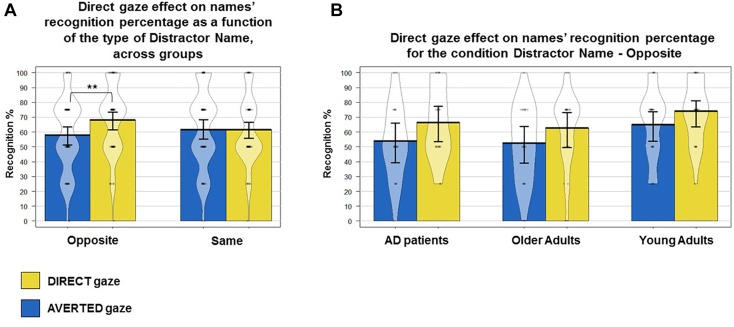
Young adults, healthy older adults and AD patients’ behavioral results. Mean, standard error to the mean, raw data and the density curve showing the full data distribution are reported. **(A)** Direct gaze effect on names recognition percentage as a function of the type of Distractor Name, across groups. An interaction between Gaze Direction and Type of Distractor Name was observed (*p* = 0.006). Significant planned comparison is indicated with ^∗∗^*p* < 0.01. **(B)** Direct gaze effect on names’ recognition percentage for the condition Distractor Name – Opposite. The effect indicated in **(A)** with ^∗∗^ is illustrated here in each group of participants (YA, OA, AD).

The ANOVA with Gaze Direction and Type of Distractor Name as within-subjects factors and Group as between-subjects factor performed on the percentage of correct Name Recognition (i.e., names correct recognitions percentage after each OLD face presentation, independently from its correct recognition ) revealed a main effect of Group (*F*_(2,62)_ = 5.28, *p* = 0.007; $\eta_{p}^{2}$ = 0.14). Planned comparisons revealed that, overall, YA (mean = 69 ± 15%) better associated names to faces than healthy OA (56 ± 16%, *t*_(43)_ = 2.68; *p* = 0.01, *d* > 0.8) and AD patients (mean = 58 ± 11%, *t*_(43)_ = 2.76; *p* = 0.008, *d* > 0.8). No differences were observed between healthy OA and AD patients (*t*_(38)_ = −0.27; *p* > 0.1). The ANOVA also revealed a trend for a gaze direction effect on this variable (*F*_(1,62)_ = 3.45 *p* = 0.06, $\eta_{p}^{2}$ = 0.05) showing that participants tended to better associate names to faces previously seen with direct (mean = 64 ± 18%) compared to averted gaze (59 ± 19%) (Figure 2C). This effect did not depend on Group (*F* < 1; *p* > 1) but on the Type of Distractor Name (*F*_(1,62)_ = 4.06, *p* = 0.04; $\eta_{p}^{2}$ = 0.06). Planned comparisons revealed that participants better associated names to faces seen with direct gaze (mean = 68 ± 23%) when confronted to a distractor name that was associated to the opposite (averted) gaze direction (mean = 57 ± 25%, *t*_(64)_ = 2.82; *p* = 0.006, *d* > 0.4), but not when confronted to a distractor name associated to the same (direct) gaze condition (mean = 61 ± 23% vs. mean = 61 ± 27%, *t*_(64)_ = −0.0; *p* = 1) (Figure 3). No triple interaction between Gaze Direction, type of Distractor Name and Group was found (all *F* < 1; all *p* > 0.1). No other main effect or interaction were observed.

#### RTs of Hits for Faces

The ANOVA with Gaze Direction as within-subjects factor and Group as between-subjects factor performed on the RT for hits for faces revealed a main effect of Group on this variable (*F*_(2,58)_ = 31.66; *p* < 0.0001; $\eta_{p}^{2}$ = 0.52). Planned comparisons showed that, overall, AD participants were slower than healthy OA (respectively, mean = 4744 ± 1325 vs. 3712 ± 1082 ms; *t*_(38)_ = 2.69; *p* = 0.01, *d* = 0.8) who were slower than YA (mean = 2094 ± 499 ms; *t*_(43)_ = 6.65; *p* < 0.0001, *d* = 2.0). No effects of Gaze Direction, or interaction between Gaze Direction and Group, were found on RT’s for Hits for Faces (all *p* > 0.1) (Table 2).

**Table 2:** Means and SDs of response times (RTs) for correct answers in the surprise recognition test for faces (RT for Hits for faces) and for names (RT for Hits for names) of AD patients, Older adults and Young adults for each experimental condition (direct/averted gaze direction).

	Young adults		Older adults		AD patients		ANOVA	Differences between groups in planned comparisons
	Direct gaze	Averted gaze	Direct gaze	Averted gaze	Direct gaze	Averted gaze		
RT for hits for faces	2242 ± 833	1984 ± 524	3734 ± 1313	3701 ± 1121	4901 ± 1621	5173 ± 2040	*F*(2,58) = 31.6^∗∗∗^	A^∗∗∗^ B^∗^
RT for hits for names	2663 ± 804	2669 ± 1202	3980 ± 1285	4049 ± 995	4947 ± 1522	5249 ± 2196	*F*(2,58) = 19.2^∗∗∗^	A^∗∗∗^ B^∗^

*SD, standard deviation; AD, Alzheimer’s disease. A: Difference between Young adults and Older adults. Two-tailed t-tests for independent samples were used. B: Difference between Older adults and AD patients. Two-tailed t-tests for independent samples were used. ^∗^p < 0.05; ^∗∗∗^p < 0.001.*

#### RTs of Hits for Name Recognition

The ANOVA with Gaze Direction as within-subjects factor and Group as between-subjects factor performed on this variable revealed a main effect of Group (*F*_(2,58)_ = 19.2; *p* < 0.0001; $\eta_{p}^{2}$ = 0.39). Planned comparisons showed that, overall, AD participants were slower than healthy OA (respectively, mean = 5161 ± 1935 vs. 4025 ± 1097 ms; *t*_(36)_ = 2.25; *p* = 0.03, *d* = 0.7) who were slower than YA (mean = 2702 ± 882 ms; *t*_(43)_ = 4.48; *p* < 0.0001, *d* = 1.9). No effects of Gaze Direction, or interaction between Gaze Direction and Group, were found (all *p* > 0.1) (Table 2).

## Discussion

Difficulty in recalling people’s names is a major concern for healthy older adults and people with AD. Previous works have shown that the use of eye contact during social interaction enhances memory for faces (Mason et al., 2004; Vuilleumier et al., 2005; Conty and Grèzes, 2011) and concomitant verbal information (Fry and Smith, 1975; Ottenson and Ottenson, 1979; Fullwood and Doherty-Sneddon, 2006). We aimed to extend our knowledge about W.E. memory effects and test whether eye contact can improve memory for Face-Name association in healthy YA, OA, and AD patients at the early to mild stage of the disease. Our results do not support this hypothesis, as direct gaze failed to increase memory for a complete face-name recognition in the three populations. Interestingly however, our data revealed a better encoding of stimuli (here faces and names) concomitantly presented with direct, as compared to averted gaze, overall on the three populations. This supports the view that eye contact context benefits memory throughout the course of aging.

Contrary to our hypothesis, the direct gaze condition did not enhance “Complete Face-Name recognition” scores in any of our groups, suggesting that eye contact does not enhance memory for Face-Name association. One critical reason could be that names do not require semantic treatment. The mere action to form face-name associations is known to be particularly difficult, owing to the inherent lack of relation between a face with a name (Werheid and Clare, 2007). By contrast, forming an association between a face and another biographical information (i.e., professions, hobbies) is easier (McWeeny et al., 1987; Cohen, 1990). McWeeny called this phenomenon the “Baker-Baker paradox”: “*[...] names remained much harder to recall than occupations. This was true even for ambiguous labels that could be used as names or as occupations. It is much harder to recall that a person’s surname is Baker than to recall that a person is a baker*” (McWeeny et al., 1987, p. 143).

The absence of any contextual properties requires indeed much more efforts and a higher level of cognitive demand to formulate an associative link, i.e., to bind a proper name to a unique face. It is thus possible that a minimal amount of semantic processing is necessary for direct gaze to benefit associative memory.

However, when focusing on the Name recognition score, our data showed that, overall, participants tended to better recognize names that have previously been presented in direct, as compared to averted gaze condition. At first view, this may have reflected a sort of covert benefit of direct gaze on face-name association. Yet, this trend was actually driven by the context of the recognition task. Participants recognized more names encoded in the direct gaze condition, only when the name was confronted to another name encoded in the averted gaze (opposite) condition. No effect was observed when the participant had to choose between two names previously encoded in the direct gaze condition. Thus, this effect actually revealed an overall benefit in encoding stimuli concomitantly presented with direct gaze (as compared to averted), instead of a benefit on associative memory. Such pattern was observed across all our groups and converges with the few existing studies that have investigated eye contact effects on memory for faces and concomitant verbal information using either teacher’s instructions (Fry and Smith, 1975), children’s tales (Ottenson and Ottenson, 1979) or sales information about fictitious product (Fullwood and Doherty-Sneddon, 2006) as to-be-remembered stimuli. In sum, if the present results do not validate the hypothesis that eye contact can enhance face-name association retrieval, they still support the view that eye contact benefits encoding capacities for concomitantly conveyed information. Yet, from a theoretical perspective, it would be interesting to further the investigation of W.E. effects on Face-Word association by varying the semantic value of the word (by using for example names vs. word related to hobbies vs. word unrelated to individuals).

Importantly also, the present data revealed a persistence of the W.E. effects on memory in normal and pathological aging. In a previous study, we suggested that the memory for faces’ effect is disrupted under the effect of aging since we only observed its emergence in YA, but not in OA and in AD patients (Lopis et al., 2017). In the present study, when improving the salience of the facial stimuli by adding contextual details (i.e., keeping the colored version of the portraits and attributing names to the individuals), we observe an overall increase of recognition performances for faces, on one hand, and names, on the other hand, previously presented with direct, as compared to averted gaze. These effects were observed across groups, suggesting that the mechanisms underlying the W.E. effects on memory may actually still be functional in healthy OA and in AD patients.

However, it is noteworthy that the overall face recognition percentage for YA, i.e., in young normal cognition, was unusually low as compared to data reported by other works (i.e., 76% in the present data vs. over 80 and 89% respectively in Hood et al., 2003 and Lopis et al., 2017 with similar sample size). So far, studies that have investigated the W.E. effects on memory for faces have asked participants: either to perform a gender identification or an age-classification task (Mason et al., 2004; Vuilleumier et al., 2005; Okruszek et al., 2017), to indicate whether the actor was physically addressing them or not (Conty and Grèzes, 2011), to simply look at the faces (Hood et al., 2003; Smith et al., 2006) or to express likability judgment over them (Lopis et al., 2017). To the best of our knowledge, the present study is the first one to propose a task requiring participants to split their attentional resources between two different kinds of stimuli (i.e., face and name). The attentional resources allocated to process face’s morphology were likely diminished. It is therefore possible that the modulation of the attentional load at the encoding stage also plays a key-role in the emergence of the W.E. memory effect in older populations. Further investigations are needed in order to clarify this question.

As expected, our results showed that, overall, AD patients were significantly slower than healthy OA who, in turn, were slower than YA in performing the two recognition tasks. These effects can be related to the general age-associated weakening of executive functions and information processing speed (for a review, see Harada et al., 2013). However, when focusing on percentage of correct response, OA performed only marginally better than AD patients on face recognition and, most importantly, did not differ from the AD patients neither on Complete Face-Name Recognition nor on Name Recognition. At a first view, this was unexpected, especially when considering that several Face-Name association memory tasks (methodologically similar to ours) have been proposed as promising tools for the early detection of cognitive deficits that may constitute early stages of AD (Rentz et al., 2011; Amariglio et al., 2012; Polcher et al., 2017). However, contradictory results have also been reported and future research should endeavor to address this issue (see Rubiño and Andrés, 2018 for a review). For example, advanced age and/or low education have also been associated with a decline of performance on these kinds of tasks (Amariglio et al., 2012; Papp et al., 2014; Sanabria et al., 2018). Plus, a revised version of the Face-Name Association Memory Exam (FNAME) – the most tested tool – has also been shown to poorly discriminate between cognitively healthy older adults and people presenting a mild cognitive impairment (MCI) (Alegret et al., 2015). In the light of these data, the lack of difference between our groups of OA and AD patients is less surprising and confirms the struggle elderly people experience in retrieving names.

## Conclusion

By investigating whether eye contact can improve memory for Face-Name association in normal aging and in AD, our study allowed to delimit the actual power of W.E. effect on memory. Our data do not support the view that W.E. can improve other’s name retrieval. However, we showed that an eye contact context can still enhance the encoding of concomitantly presented stimuli (here face and name independently). Further, our results are the first empirical evidence that the W.E. memory effect are preserved in normal aging and in AD. However, further investigations are needed to elucidate the conditions that favor the emergence of the W.E. effect on memory in older people.

## Ethics Statement

All participants provided written informed consent according to institutional guidelines of the local research ethics committee (who stated on the compliance with the Declaration of Helsinki). The whole procedure was approved by the local ethics committee (CPP IdF-X, protocole 02(2) 2015 – ALCOM No. 2014-A01141-46).

## Author Contributions

LC and DL conceived and designed the experiments and wrote the manuscript. DL collected the data and performed the analysis.

## Conflict of Interest Statement

The authors declare that the research was conducted in the absence of any commercial or financial relationships that could be construed as a potential conflict of interest.

## Footnotes

1 https://www.insee.fr/fr/statistiques/3532172
